# The Effects of Feature-Based Priming and Visual Working Memory on Oculomotor Capture

**DOI:** 10.1371/journal.pone.0142696

**Published:** 2015-11-13

**Authors:** Jeroen D. Silvis, Artem V. Belopolsky, Jozua W. I. Murris, Mieke Donk

**Affiliations:** Department of Cognitive Psychology, Vrije Universiteit Amsterdam, Amsterdam, The Netherlands; University of Verona, ITALY

## Abstract

Recently, it has been demonstrated that objects held in working memory can influence rapid oculomotor selection. This has been taken as evidence that perceptual salience can be modified by active working memory representations. The goal of the present study was to examine whether these results could also be caused by feature-based priming. In two experiments, participants were asked to saccade to a target line segment of a certain orientation that was presented together with a to-be-ignored distractor. Both objects were given a task-irrelevant color that varied per trial. In a secondary task, a color had to be memorized, and that color could either match the color of the target, match the color of the distractor, or it did not match the color of any of the objects in the search task. The memory task was completed either after the search task (Experiment 1), or before it (Experiment 2). The results showed that in both experiments the memorized color biased oculomotor selection. Eye movements were more frequently drawn towards objects that matched the memorized color, irrespective of whether the memory task was completed after (Experiment 1) or before (Experiment 2) the search task. This bias was particularly prevalent in short-latency saccades. The results show that early oculomotor selection performance is not only affected by properties that are actively maintained in working memory but also by those previously memorized. Both working memory and feature priming can cause early biases in oculomotor selection.

## Introduction

Attended stimuli are typically remembered better than those that are ignored. This simple fact suggests that visual attention serves as a gateway to memory. Interestingly, however, Desimone and Duncan [1] pointed out that the relationship between attentional selection and memory is bi-directional. Memory mechanisms can be a key factor in determining which stimuli are attended. Memorized items may gain a competitive advantage relative to information that is not represented in visual working memory (VWM). Desimone and Duncan [1] reasoned that this is due to the strong overlap in structure and functionality between selection and memory mechanisms [1–7].

A number of studies found evidence suggesting that VWM content automatically affects attentional [8, 9] and oculomotor selection [10–13]. For instance, Hollingworth et al. [10] employed an oculomotor task that was embedded in a memory task. Participants held a specific color in memory while shifting gaze towards predefined target objects. Participants were asked to saccade as quickly and accurately as possible. The results demonstrated that eye movements were significantly faster and more precise when the color of the saccadic target happened to match the color held in VWM. Furthermore, when a distractor object was included in the target display, a competitive advantage was observed for the memory-matching color. Saccades were directed closer towards the distractor or target, depending on which of these objects was congruent with the color held in memory. Importantly, these effects were found even at very fast saccadic latencies (<150 ms), suggesting that VWM is capable of affecting those eye movements that are typically assumed stimulus-driven rather than goal-driven (e.g., [14–17]). Hence, Hollingworth et al. [10] concluded that maintenance of information in VWM leads to automatic modulation of early sensory processes, enhancing the perceptual salience of features held in VWM.

To ensure that their results were driven by active VWM maintenance, rather than by feature priming [18], Hollingworth et al. [10] conducted a control experiment in which participants were asked to merely observe a certain color without keeping it in memory. The results showed that the match between the previewed color and the color of the target did not have any effect on either saccade latencies or on accuracy of saccade landing positions. Based on these results, Hollingworth et al. [10] concluded that the early effects of color matches, as reported in their main experiments, were solely driven by active maintenance of color in VWM and not by feature priming. However, it is questionable whether the passive observation of a color was sufficient to evoke feature priming. Several studies point out that feature priming critically depends on prior active visual selection and plain repetition of a feature over consecutive trials is not sufficient for feature priming to occur [19–22].

The aim of the current study was to examine whether early memory effects on oculomotor selection are indeed mediated by the active maintenance of features in VWM or by the selection history of particular features; feature-based priming. Two experiments combining a search task with a memory task were closely modeled after the study of Hollingworth et al. [10]. In the search task, participants were asked to saccade to a target object that was simultaneously presented with a distractor object. Both objects were line segments of different orientations (e.g. target left tilted, distractor right titled), and were given a task-irrelevant color which varied from trial to trial. In the memory task, observers were asked to remember a specific color. The color held in memory could match the target (target-match), match the distractor (distractor-match), or match none of the objects in the search task (no-match). Participants were explicitly told that the colors presented in the search task were not in any way relevant to the task objectives. In Experiment 1, as in Hollingworth et al. [10], the memory color had to be maintained in memory throughout the search task. However, in Experiment 2, the memory for color was tested prior to the search task, eliminating the need to actively maintain the color in VWM during the search task. Importantly, in both experiments, the memory task was identical and required participants to actively select the to-be-remembered color prior to the search task. To examine the influence of VWM content and feature priming on oculomotor selection, we measured the proportions of saccades that were correctly directed towards the target in the different conditions while taking into account possible differences in saccadic latencies.

If rapidly generated saccades are driven by VWM content, short latency saccades should be more often directed to the object that matches the memorized color than to the non-matching object. This should only be evident in Experiment 1 which requires active maintenance of feature information in memory, but not in Experiment 2. If, on the contrary, early oculomotor biases are the result of feature priming, short latency saccades should be affected by a color match in both experiments to an equal extent. Finally, if early oculomotor biases are driven by both VWM maintenance and priming, the effect of a color match on rapidly generated saccades should be stronger in Experiment 1 than in Experiment 2.

## Experiment 1

### Method

#### Participants

Fourteen paid volunteers (10 female) between 19 and 24 years of age participated in the experiment which was conducted at the Vrije Universiteit Amsterdam. All participants reported to have normal or corrected-to-normal vision, and none reported to be colorblind. The experiment was conducted in accordance with the guidelines of the Helsinki Declaration. Participants signed a written informed consent before they took part in the study. The study was approved by the scientific and Ethical Review Committee (VCWE), an independent advisory board of the Faculty of Psychology and Education (FPP) at the Vrije Universiteit Amsterdam.

#### Apparatus

The experiment was conducted in a sound-isolated and dimly lit room. Participants were asked to position their chin on a chinrest which was installed at a distance of 75 cm from a 21-inch Samsung Syncmaster display (100 Hz) with a resolution of 1680 x 1050 pixels. The computer had an Intel Core 2 Duo (3 Ghz) processor and a NVIDEA GeForce 210 video card. The task was programmed using the experiment-builder software OpenSesame [23]. Manual responses were given through a QWERTY keyboard. Monocular movements were recorded with an Eyelink 1000 (Desktop Mount model, infra-red video-based, SR Research Ltd., Canada), with a resolution of 1000 Hz (temporal) and 0.01° RMS (spatial). The eye tracker additionally contained an eye illuminator and was placed at a distance of approximately 50 cm from the chinrest. It classified eye movements surpassing a 35°/s velocity or a 9500°/s² acceleration as saccadic movements.

#### Design, stimuli, and procedure

**Fig 1:**
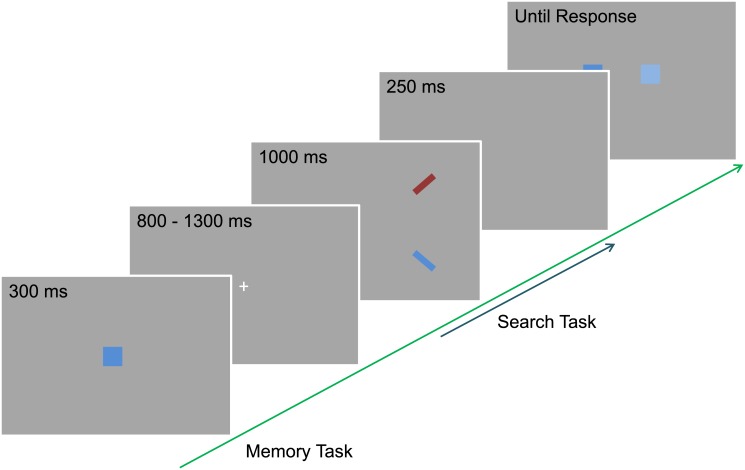
The design of the trial. The search task was performed during the maintenance interval of the memory task. In this particular example, the match between the memory and search task is exact, but if it was not exact, the one color presented in the search task would match the false alternative in the response display of the memory task.

Each trial comprised two tasks, a memory task and a search task (see Fig 1). To start a trial participants fixated a central point on the screen and pressed the spacebar key, activating a drift correction. Immediately after the drift correction, a colored square (1.6°) was presented at the center of the screen for 300 ms and participants had to memorize its exact color and keep it in memory until the end of the trial. On each trial the color of the square was equally likely to be chosen from three main colors (red, green, and blue). The exact shade of the presented color was determined randomly from four different alternatives (see appendix). The memory square was followed by a fixation dot which was presented for a random duration between 800 and 1300 ms [24].

After this period elapsed, the target and the distractor objects for the search task were presented for a duration of 1000 ms, regardless of the speed of the participants' response. Both objects were line segments (1.0° x 0.2°) and were tilted 45° in the opposite direction. For example, if the target was a left-tilted line then the distractor was a right-tilted line. This relationship was kept constant throughout the experiment and counterbalanced across participants. Participants were instructed to make a speeded eye movement towards the target object of a certain orientation. The positions of the target and distractor objects varied from trial to trial and were randomly chosen from four possible locations, one in each quadrant (45°, 135°, 225°, 315°) and each 6° from the fixation.

There were three conditions in the search task: (i) the target matched the memorized color (target-match), (ii) the distractor matched it (distractor-match), (iii) or none of the objects matched the memorized color (no-match). If an object matched the memorized color, it was equally likely to be either an exact match, or a non-exact match. In case of a non-exact match, the color shade was different from the memorized shade and randomly selected from the three remaining shades of the corresponding color. This color later served as the false alternative in the memory test display. This ultimately served to prevent a potential strategy of participants to exploit the search task to improve memory performance (as in Hollingworth et al. [10]). If an object did not match the memorized color, it was equally likely to be selected from the remaining colors and shades.

Following 250 ms after the search task, a memory test display appeared. It contained two squares (1.6°) horizontally aligned in the middle of the screen separated by 2.5°. One of the squares had the exact color held in memory and the other was a different shade of the same color. The presentation of this test display was terminated by a button press of the participant to indicate which of the squares consisted of the memorized shade, the ‘n’ or ‘m’ key respectively referring to the left and the right colored square.

The experiment consisted of 1 practice block and 12 experimental blocks, each comprising 36 trials, leading to a total of 432 trials. All conditions occurred equally often in each block of trials, i.e., the target-match, the distractor-match, and the no-match condition. Task instructions of both tasks were displayed at the beginning of each block. At the end of each block feedback was provided regarding the average saccade latency and the average memory accuracy. At the end of the practice block, the average accuracy in the search task was also added to the feedback display. To train participants on the search task a high pitch tone signaled when distractor was fixated instead of the target.

### Results

Trials with first saccades faster than 50 ms and slower than 500 ms (9.1%), saccades that did not start within 1.5° away from fixation point (1.1%), and saccades that did not land within 1.5° of the target or distractor (17.0%) were discarded from further analyses. This resulted in an average loss of approximately 27.2% of trials. This substantial loss of data was likely related to the difficulty of performing the two tasks simultaneously (for comparison see Experiment 2, in which the tasks were performed sequentially). The exclusion left approximately 314 observations per condition per participant, which still represents a sufficiently large number for further analyses. A trial was denoted correct when the first eye movement landed within 1.5° of the target.

To examine the time course of oculomotor selection, all trials were rank-ordered by latency across conditions but separately for each participant. Subsequently, the trials were divided into four equal bins. Next, for each of these bins the proportions of correct responses were separately calculated per condition and participant. This approach allowed us to compare the proportions of correct responses across the conditions, while taking into account possible differences in latency (see e.g. Siebold and Donk [25], and Siebold, van Zoest [26] for a similar approach)

**Fig 2:**
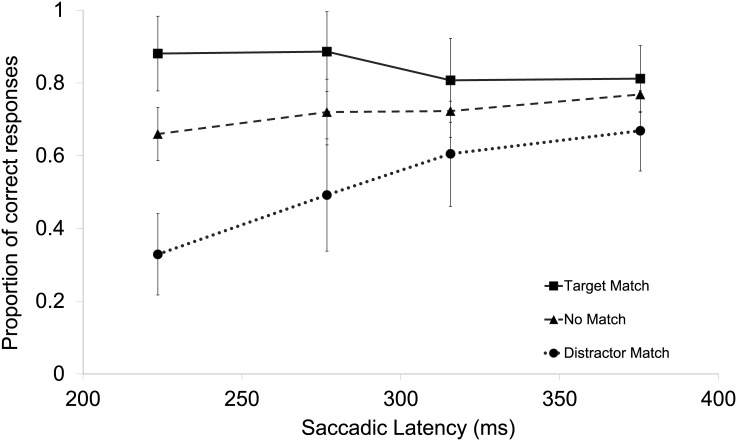
The main results of Experiment 1. Illustrated are the mean proportions of correct responses in the search task separately per condition and latency bin. The error bars reflect the within-subject 95% confidence interval of the means calculated and corrected separately per bin [27, 28].

A repeated-measures ANOVA was performed on the individual proportions of correct responses in the search task with Color Match (target-match, distractor-match, no-match) and Latency bin (1–4) as factors. The results showed a main effect of Color Match, *F*(2,26) = 15.77, *p* < .001, $\eta_{p}^{2}$ = .548, a main effect of Latency bin, *F*(3,39) = 5.61, *p* = .003, $\eta_{p}^{2}$ = .301, and a significant Color Match x Latency bin interaction, *F*(6,78) = 8.30, *p* < .001, $\eta_{p}^{2}$ = .390 (see Fig 2). The results of the ANOVA indicate that the effect of Color Match declines over time. Indeed, when comparing the target-match and distractor-match conditions, there is only a significant difference between the first two latency bins (respectively *t*(13) = 9.315, *p* < .001, and *t*(13) = 3.681, *p* = .003), and not between the last two (respectively *t*(13) = 1.872, *p* = .084, and *t*(13) = 1.825, *p* = .091.

**Fig 3:**
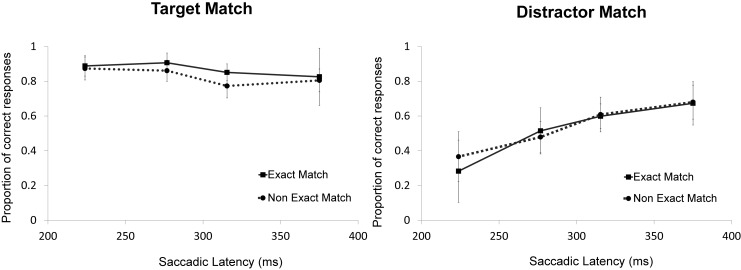
Exact versus non-exact match in Experiment 1. Illustrated are the mean proportions of correct responses separately per latency bin and shade match in the search task separately plotted for the target-match and distractor-match conditions. The error bars reflect the within-subject 95% confidence intervals of the means calculated and corrected separately per bin.

To examine whether there was any difference between an exact memory match and a non-exact memory match, an ANOVA was performed on the individual proportions of correct responses with the factors Color Match (target-match, distractor-match), Shade Match (exact, non-exact), and Latency bin (1–4). The results showed a main effect of Color Match, *F*(1,13) = 17.28, *p* = .001, $\eta_{p}^{2}$ = .571, no effect of Shade Match, *F*(1,13) < 1, and a main effect of Latency bin, *F*(3,39) = 6.24, *p* = .001, $\eta_{p}^{2}$ = .324. Moreover, there was a Color Match x Shade Match interaction, *F*(1,13) = 4.69, *p* = .049, $\eta_{p}^{2}$ = .265, which suggests that exact memory matches affected oculomotor selection more profoundly than non-exact matches, but only when the target was the matching object. Furthermore, there was a Color Match x Latency bin interaction, *F*(3,39) = 11.83, *p* < .001, $\eta_{p}^{2}$ = .476, and no Shade Match x Latency bin interaction, *F*(3,39) = 1.11, *p*>.25. Most importantly, there was no three-way (Color Match x Shade Match x Latency bin) interaction, *F*(3,39) < 1. The Color Match x Latency bin effect appeared to be driven by the colors rather than the shades of the colors (see Fig 3).

An ANOVA performed on the individual means of the saccadic latencies with the factor Color Match (target-match, distractor-match, no-match) showed a main effect of Color Match, *F*(2,26) = 12.16, *p* < .001, $\eta_{p}^{2}$ = .483. Latencies in the distractor-match condition (*M* = 297 ms) were higher than in the target-match condition (*M* = 289 ms), *F*(1,13) = 4.799, *p* = .047, $\eta_{p}^{2}$ = .270, but were lower than the no-match condition (*M* = 311 ms), *F*(1,13) = 18.04, *p* = .001, $\eta_{p}^{2}$ = .581. A similar ANOVA performed on only those trials that yielded a correct response showed similar results. There was a main effect of Color Match, *F*(2,26) = 9.859, *p* < .001, $\eta_{p}^{2}$ = .431. Latencies in the distractor-match condition (*M* = 314 ms) were higher than in the target-match condition (*M* = 287 ms), *F*(1,13) = 12.328, *p* < .005, $\eta_{p}^{2}$ = .487, but were similar to the no-match condition (*M* = 317 ms), *F*(1,13) = .163, *p*>.25.

Finally, the proportions of correct responses in the memory task (see Table 1) were unaffected by Color Match (target-match, distractor-match), *F*(1,13)< 1, and Shade Match (exact, non-exact), *F*(1,13)< 1, nor was there a Color Match x Shade Match interaction, *F*(1,13)< 1. Hence, performance in the memory task was not affected by color and shade match. In the no-match condition, memory performance was 83.7% (*SE* = 1.1%). To examine whether memory performance was related to the strength of the color match effect in the search task, an ANOVA was performed on the proportions of correct responses in the search task using the factors Color Match (target-match, distractor-match, no-match) and Memory Performance (correct, incorrect). The results showed a main effect of Color Match, *F*(2, 26) = 16.770, *p* < .001, $\eta_{p}^{2}$ = .563, no effect of Memory Performance, *F*(1,13) = .053, *p*>.25, and no interaction between Color Match and Memory Performance, *F*(2,26) = 1.180, *p*>.25. These findings suggest that a failure to maintain a specific color representation does not weaken the strength of the color match effect. Apparently, the color match effect is mainly driven by color category rather than the exact shade of the color representation.

**Table 1:** Memory task performance. Mean proportions of correct responses in the memory task for each color match and shade match condition with values between brackets representing standard errors of the mean per condition.

		Shade Match	
Color Match		Exact	Non-exact
	Target-match	86.0% (1.3%)	82.9% (2.3%)
	Distractor-match	83.3% (1.3%)	83.5% (1.8%)

### Discussion

The results demonstrated that holding a color in working memory biased the oculomotor selection of similar colors in the search display. Saccades were more likely to be directed to the objects matching the color held in working memory, irrespectively of whether these objects were defined as targets or distractors. An interesting detail within these results is that the working memory influence appeared most profound when the search target's color was an exact rather than non-exact match with the memory color, whereas no accuracy difference between exact and non-exact matches was observed for the distractor object. One possible interpretation is that participants were more sensitive to color shade differences of the target than differences of the distractor object because participants attempted to ignore the distractor. Alternatively, it might be an incidental finding because the effect was barely significant (*p* = .049) and no such effects were observed in Hollingworth et al. [10]or in Silvis and Van der Stigchel [12].

Furthermore, the effect of VWM content was especially pronounced early in time (<250 ms) and diminished as the saccade latency increased. The transient time course of this effect is reminiscent of the effect of salience on visual selection [15]. Note, however, that in the present experiment both objects were equally salient (in a physical sense) and the early bias in the oculomotor selection was driven by the match to the information held in working memory. Furthermore, the match to working memory content also speeded up selection with saccades being initiated faster to the matching objects. The present results are consistent with the idea that memorized content automatically modulates early sensory processes by enhancing the relative salience of those stimuli that matched the color held in VWM [10].

However, it is unclear whether these transient effects were truly mediated by the active maintenance of VWM content. The results of Experiment 1 can also be explained by feature priming [18, 29–32]. Specifically, the selection of the to-be-remembered color prior to the search task might have been sufficient to bias the subsequent selection process towards the memorized color. This possibility was examined in Experiment 2. The tasks and stimuli were kept exactly the same as in Experiment 1 except that the memory task was completed before the start of the search task. That is, participants were not required to hold a color in memory during the search task.

## Experiment 2

### Method

#### Participants

Fourteen paid volunteers (10 female) between 19 and 26 years of age participated in Experiment 2 which was conducted at the Vrije Universiteit Amsterdam. All participants reported to have normal or corrected-to-normal vision, and none reported to be colorblind. The experiment was conducted in accordance with the guidelines of the Helsinki Declaration. Participants signed a written informed consent before they took part in the study. The study was approved by the scientific and Ethical Review Committee (VCWE), an independent advisory board of the Faculty of Psychology and Education (FPP) at the Vrije Universiteit Amsterdam.

#### Design, stimuli, and procedure

The design, procedure, and stimuli employed in Experiment 2 were similar to Experiment 1 except that the memory task was completed prior to the start of the search task.

Each trial started with the drift correction, followed by the memory task consisting of the presentation of the colored square (300 ms) and a blank interval of 700 ms. Thereafter, the memory test display appeared containing the two colored squares, one of which matched the memorized color. After the response of the participant, a central fixation dot was presented for a duration between 800 and 1300 ms, after which the search task, identical to the one used in Experiment 1, started.

### Results

Trials with first saccades faster than 50 ms and slower than 500 ms (3.1%), saccades that did not start within 1.5° away from fixation point (0.1%), and saccades that did not land within 1.5° from the target or distractor (10.0%) were discarded from further analyses. This resulted in an average loss of approximately 14.7% of trials.

**Fig 4:**
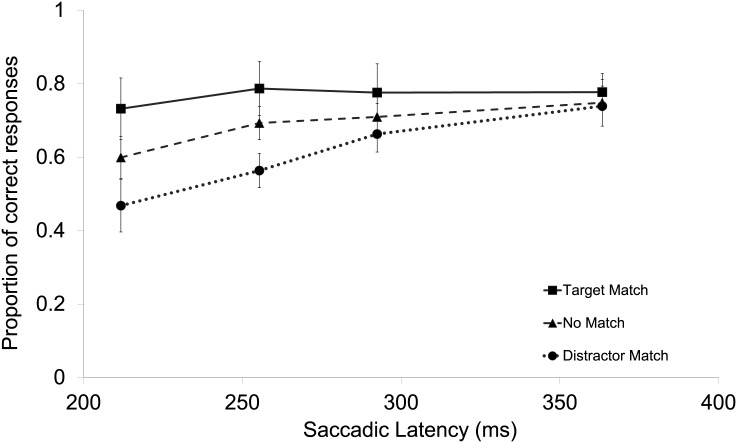
The main results of Experiment 2. Illustrated are the mean proportions of correct responses plotted for each condition and latency bin. The error bars reflect the within-subject 95% confidence intervals of the means calculated and corrected separately per bin.

A repeated-measures ANOVA was performed on the individual proportions correct responses in the search task with Color Match (target-match, distractor-match, no-match) and Latency bin (1–4) as factors. The results showed a main effect of Color Match, *F*(2,26) = 34.91, *p* < .001, $\eta_{p}^{2}$ = .73, a main effect of Latency bin, *F*(3,39) = 15.82, *p* < .001, $\eta_{p}^{2}$ = .549, and a significant Color Match x Latency bin interaction, *F*(6,78) = 3.98, *p* = .002, $\eta_{p}^{2}$ = .234. This pattern of results indicates that the effect of Color Match declines over time (see Fig 4). When comparing the target-match and distractor-match conditions, the first three latency bins differ significantly (respectively, *t*(13) = 5.070, *p* < .001, *t*(13) = 4.792, *p* < .001, and *t*(13) = 2.309, *p* = .038), whereas for the last latency bin no difference was found (*t*(13) = 1.198, *p* = .252).

**Fig 5:**
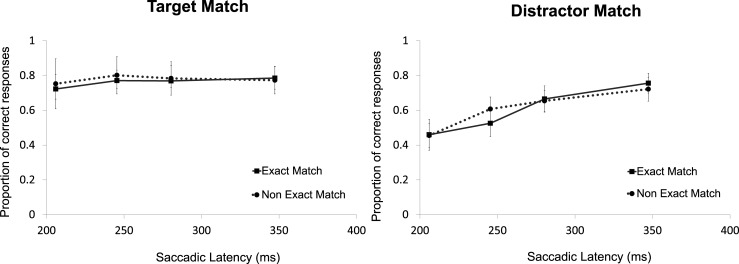
Exact versus non-exact match in Experiment 2. Illustrated are the mean proportions of correct responses separately per latency bin and shade match in the search task separately plotted for the target-match and distractor-match conditions. The error bars reflect the within-subject 95% confidence intervals of the means calculated and corrected separately per bin.

To examine whether there was a difference between an exact memory match and a non-exact memory match, an ANOVA was performed on the individual proportions of correct responses using the factors Color Match (target-match, distractor-match), Shade Match (exact, non-exact), and Latency bin (1–4). The results show a significant main effect of Color Match, *F*(1,13) = 52.46, *p*<001, $\eta_{p}^{2}$ = .801, no effect of Shade Match, *F*(1,13)< 1, and an effect of Latency bin, *F*(3,39) = 15.88, *p* < .001, $\eta_{p}^{2}$ = .550. Furthermore, there was no Color Match x Shade Match interaction, *F*(1,13)< 1, a significant Color Match x Latency bin interaction, *F*(3,39) = 5.18, *p* = .004, $\eta_{p}^{2}$ = .285, and no Shade Match x Latency bin interaction, *F*(3,39) = 1.16, *p* = .338. Most importantly, there was no three-way (Color Match x Shade Match x Latency bin) interaction, *F*(3,39)< 1 (See Fig 5).

An ANOVA performed on the mean individual saccadic latencies showed no significant effect of Color Match, *F*(2,26)< 1. The mean saccadic latency was 270 ms.

#### Comparison between experiment 1 and 2

To investigate whether there is any difference between Experiment 1 and 2, a mixed-effects ANOVA was performed on the proportions of correct oculomotor responses with Experiment as a between-subjects factor and Color Match (target-match, distractor-match, no-match) and Latency bin (1–4) as the within-subjects factors. The results showed a main effect of Color Match, *F*(2,52) = 31.51, *p* < .001, $\eta_{p}^{2}$ = .548, which interacted with Experiment, *F*(2,52) = 3.79, *p* = .029, $\eta_{p}^{2}$ = .127. Also a main effect of Latency bin was shown, *F*(3,78) = 18.19, *p* < .001, $\eta_{p}^{2}$ = .412, but Latency bin did not interact with Experiment, *F*(3,78)< 1. There was a Color Match x Latency bin interaction, *F*(6,156) = 11.99, *p* < .001, $\eta_{p}^{2}$ = .613. Finally, and most importantly, there was no Experiment x Color Match x Latency Bin interaction, *F*(6,156) = 1.23, *p* = .292.

A mixed-effects ANOVA on the saccadic latencies showed a main effect of Color Match, *F*(2,52) = 8.634, *p* = .001, $\eta_{p}^{2}$ = .249, and a Color Match x Experiment interaction, *F*(2,52) = 11.856, *p* < .001, $\eta_{p}^{2}$ = 313, indicating that the main effect of Color Match on latency was larger in Experiment 1 than in Experiment 2 (see Figs 1 and 2).

Performance in the memory task was better in Experiment 2 (*M* = 89.6%) than in Experiment 1 (*M* = 83.9%), *t*(26) = 3.51, *p* = .002, indicating that it was easier to perform the memory task prior to the search task than simultaneously with the search task.

### Discussion

The results of Experiment 2 showed that even when there was no need to actively maintain color information in VWM, early oculomotor selection behavior was affected by the color previously selected for the memory task. The interaction between Color Match and Latency bin was similar to the one observed in Experiment 1 suggesting that feature priming underlies the early transient modulations in oculomotor selection. Note that the word transient refers to the time course of oculomotor selection on a given trial (as reflected in the decreasing effect of Color Match with increasing saccadic latency).

Even though the interaction between Color Match and Latency Bin was similar across experiments, the main effect of Color Match was larger in Experiment 1 than in Experiment 2. This shows that the active maintenance of a memory representation as it was required in Experiment 1, but not in Experiment 2, amplifies the selection bias equally strongly across all latency bins. Finally, no effect was observed for the type of color match. When the match between the memory color and a color in the search task was exact, the match effect was equally strong compared to a match that was not exact. This was true for a target and for a distractor match. A reason as to why a match type effect was observed in Experiment 1 may relate to the involvement of working memory. However, as was mentioned before, it could have been an incidental finding as the effect was barely significant (*p* = .049) and also not observed in Hollingworth et al. [10] and Silvis and Van der Stigchel [12].

Although we attribute the differences between Experiment 1 and 2 to working memory mechanisms, one must take into account that the absence of a need to employ working memory during search in Experiment 2 does not necessarily rule out the possibility that working memory was still involved to a certain extent in the search task. It is possible that participants held on to a color representation throughout each trial. However, there are reasons to believe that this is unlikely. First, the experiments were designed to eliminate performance benefits if colors were used strategically across the two subtasks. If participants attempted to use the memory color to boost search performance, they would find that only in a third of the trials the target indeed matched the memory color, and only in one sixth of the trials this match was exact. Second, working memory maintenance is demanding. It takes effort to maintain specific content in working memory. Moreover, previous studies using a similar procedure (e.g., [9, 33]) typically found no matching effects when the memory test had been completed before the search task showing that participants are usually not inclined to keep items of a prior memory task into active working memory. Since there was enough time to drop the color representation from working memory [34, 35], the only plausible motivation of a participant to keep a color representation active would have been a misinterpretation of the task instructions.

## General Discussion

Together, the experiments show that oculomotor selection is affected by both feature priming and VWM content. The effects of feature priming are transient and thus most pronounced in early selection. In fact, the effects of feature-based priming had already vanished in the last two latency bins. The effects of VWM content are more sustained. Color match affected oculomotor selection more strongly in Experiment 1 than in Experiment 2, and this effect was present in all latency bins. Importantly, the increase in the strength of the match effect, as induced by active maintenance of VWM content, is equally large across all latency bins. This implies that VWM compliments the effects of priming thoroughly, but it does not alter the nature of the priming effect. The present results are in line with earlier findings showing that VWM content affects even the most rapid eye movements [10]. Different from previous studies is our finding of a (transient) color match effect in the search task without active memory maintenance, i.e. when participants were not required to actively hold a color in memory. Feature priming should thus be taken as a more important factor than previously assumed [36–38]. The current findings confirm the automatic nature of the color match effects. The selection bias rapidly declines with saccadic latency: a time course that is characteristic for automatic selection [15, 39–43].

The current work is not the first to examine feature priming as an account for biases observed in oculomotor selection. Examples are Hollingworth, et al. [10] and Silvis and Van der Stigchel [12] who also attempted to rule out priming as an alternative account. However, instead of performing a VWM memory task, in these studies participants were asked to simply observe a color prior to the eye movement task. Unfortunately, merely observing a color does not guarantee that the color is actually selected by attention, and so not finding any effects cannot rule out the potential role of feature priming [19–22].

There are also other important differences between Hollingworth et al. [10] and the current work. In Hollingworth et al. [10] there was absolutely no ambiguity about which object was the target and which object was the distractor. The distractor (if it appeared) was always presented at a location where the target could never appear. Also the sizes of the target and the distractor were distinct. Consequently, Hollingworth et al. [10] investigated accuracy in terms of saccadic amplitude and in terms of landing distance relative to the target object, rather than the dichotomous correct/incorrect target selection classification as used in the current experiments. Whether this may also explain differences in the role of feature-based priming remains unclear and needs further examination.

There are examples in literature, however, that are more difficult to reconcile with the current findings [9, 33]. In these studies, participants were asked to memorize a color and search for a target diamond presented among distractor circles. One of these distractors could have a distinct color that could either match or mismatch the color held in memory. As in the present study, the memory for color was tested either before or after the search task. However, search performance was measured with manual rather than oculomotor responses. The results showed that when memory was tested before the search task, there was no difference in search speed between trials involving a matching or mismatching distractor color, whereas there was a difference when memory was tested after the search task (i.e., when items had to be actively maintained in VWM). These results suggest that feature priming does not affect visual selection. Alternatively it is possible that the absence of an effect of priming in Olivers and Eimer [33] and in Olivers et al. [9] was related to the fact that they used manual responses rather than oculomotor responses: manual responses are generally much slower than oculomotor responses. Perhaps the manual responses in these studies were too slow to exhibit the rapidly declining effects of priming.

Overall, in the present study we demonstrate that feature priming can underlie the color match effects observed in prior VWM studies. Perhaps the effects of feature priming have not been observed in prior work because they are transient and only present for fast responses. With active memory maintenance, people exhibit a more sustained bias towards the memorized color which does not vary with saccadic latency. This may suggest that feature-based priming by itself suffices to induce memory match effects, but this effect of priming is fundamentally bolstered by active memory maintenance.

## Appendix

**Table:** 

Category										
		Green			Red			Blue		
		X	Y	cd/m^2^	X	Y	cd/m^2^	X	Y	cd/m^2^
Shades	1	0.43	0.41	25.1	0.54	0.29	43.8	0.17	0.14	29.4
	2	0.32	0.49	30.0	0.57	0.32	43.0	0.19	0.13	25.1
	3	0.24	0.47	28.2	0.63	0.31	39.4	0.20	0.15	30.2
	4	0.26	0.36	29.5	0.54	0.31	38.7	0.22	0.15	28.4

The CIE x, y coordinates and luminance values of all shades of the three color categories used in the experiment (measured with a TEK Lumacolor J17 photometer).

## Supporting Information

S1 Dataset Experiment 1. (CSV)

S2 Dataset Experiment 2. (CSV)
